# Multi-Class Malocclusion Detection on Standardized Intraoral Photographs Using YOLOv11

**DOI:** 10.3390/dj14010060

**Published:** 2026-01-16

**Authors:** Ani Nebiaj, Markus Mühling, Bernd Freisleben, Babak Sayahpour

**Affiliations:** 1 Department of Orthodontics, Johann-Wolfgang Goethe University, 60596 Frankfurt am Main, Germany; 2 Department of Mathematics & Computer Science, Marburg University, 35043 Marburg, Germany

**Keywords:** artificial intelligence, clinical decision support systems, computer vision, deep learning, dental photography, intraoral photographs, malocclusion, multi-view imaging, object detection, orthodontics

## Abstract

**Background/Objectives:** Accurate identification of dental malocclusions from routine clinical photographs can be time-consuming and subject to interobserver variability. A YOLOv11-based deep learning approach is presented and evaluated for automatic malocclusion detection on routine intraoral photographs, testing the hypothesis that training on a structured annotation protocol enables reliable detection of multiple clinically relevant malocclusions. **Methods:** An anonymized dataset of 5854 intraoral photographs (frontal occlusion; right/left buccal; maxillary/mandibular occlusal) was labeled according to standardized instructions derived from the Index of Orthodontic Treatment Need (IOTN) A total of 17 clinically relevant classes were annotated with bounding boxes. Due to an insufficient number of examples, two malocclusions (transposition and non-occlusion) were excluded from our quantitative analysis. A YOLOv11 model was trained with augmented data and evaluated on a held-out test set using mean average precision at IoU 0.5 (mAP50), macro precision (macro-P), and macro recall (macro-R). **Results:** Across 15 analyzed classes, the model achieved 87.8% mAP50, 76.9% macro-P, and 86.1% macro-R. The highest per-class AP_50_ was observed for Deep bite (98.8%), Diastema (97.9%), Angle Class II canine (97.5%), Anterior open bite (92.8%), Midline shift (91.8%), Angle Class II molar (91.1%), Spacing (91%), and Crowding (90.1%). Moderate performance included Anterior crossbite (88.3%), Angle Class III molar (87.4%), Head bite (82.7%), and Posterior open bite (80.2%). Lower values were seen for Angle Class III canine (76%), Posterior crossbite (75.6%), and Big overjet (75.3%). Precision–recall trends indicate earlier precision drop-off for posterior/transverse classes and comparatively more missed detections in Posterior crossbite, whereas Big overjet exhibited more false positives at the chosen threshold. **Conclusion:** A YOLOv11-based deep learning system can accurately detect several clinically salient malocclusions on routine intraoral photographs, supporting efficient screening and standardized documentation. Performance gaps align with limited examples and visualization constraints in posterior regions. Larger, multi-center datasets, protocol standardization, quantitative metrics, and multimodal inputs may further improve robustness.

## 1. Introduction

Intraoral photography is routine in dental diagnostics, documentation, and follow-up because it is radiation-free, inexpensive, and easy to standardize across visits [1]. However, turning photographs into consistent diagnostic labels remains susceptible to inter- and intra-observer variability, which can influence treatment planning and outcomes [2,3]. Recent validation work shows that photographic assessments can be clinically acceptable but still exhibit only moderate-to-substantial human rater agreement, underscoring the need for standardized, reproducible pipelines [4].

In orthodontics, deep learning on intraoral photographs has so far been dominated by narrow task definitions (single traits or limited label sets). For example, models have been proposed for crowding categorization and for predicting treatment-related labels from photographs, yet such outputs must be interpreted as supportive signals rather than substitutes for comprehensive diagnosis, because treatment planning (including extraction decisions) is inherently multimodal [5]. Beyond classification, object detection can add spatial localizations that may facilitate clinical interpretation and quality assurance by highlighting image regions consistent with a predicted finding, and this approach has been applied to localize malocclusion traits such as crossbite on clinical photographs [6]. Recent work also demonstrated photo-based quantification of occlusal crowding and detection of surface changes (e.g., white spot lesions), supporting the broader premise that standardized intraoral photos can enable scalable, automated phenotyping beyond purely categorical outputs [7,8]. Efforts toward multi-view, multi-condition datasets are an important step toward benchmarking, but available resources still differ substantially in capture protocols, labeling granularity, and class balance [9].

Despite this momentum, reviews converge on persistent methodological gaps: predominantly single-center cohorts; small sample sizes; heterogeneous lighting, retraction, and magnification; limited external validation; and incomplete transparency around labeling rules and failure analyses [10,11]. These observations suggest detectors that (i) produce clinically interpretable spatial localizations, (ii) operate across standard intraoral views, and (iii) follow explicit, reproducible labeling guidelines grounded in accepted orthodontic criteria.

Deep learning-based object detection models, particularly current one-stage architectures of the YOLO family, enable image-level localization and multi-class prediction in a single pass [12,13]. For orthodontic photography, this could standardize assessments while preserving a radiation-free, low-cost workflow suitable for triage and longitudinal monitoring. However, few studies report multi-class, multi-view detection with explicit, clinically interpretable bounding box definitions spanning a broad malocclusion set, and even fewer publish detailed failure mode analyses or reproducibility documentation.

YOLOv11 is a state-of-the-art object detection approach with an advanced convolutional neural network architecture, an optimized detection head for precise bounding box regression, and maximized efficiency while maintaining high accuracy that provides a large deployment flexibility for hardware with less computing power, especially for CPU-based inference or edge computing.

Building on this rationale, we present and evaluate a YOLOv11-based object detector to localize and identify 15 clinically relevant malocclusion categories on routine intraoral views (frontal, right/left buccal, maxillary, and mandibular occlusal). We hypothesize that training on explicit, reproducible annotation guidelines enables reliable multi-class detection on real-world photographs, supporting standardized screening and documentation.

## 2. Materials and Methods

The study started in May 2024 with the concept phase. Administrative and regulatory preparation was conducted approximately between August and September 2024, with the data protection vote granted on 13 August 2024. Ethics approval was obtained in November 2024 (protocol code 2024-2071; approval date 12 November 2024). Following ethics committee approval, data acquisition for this retrospective, single-center diagnostic study was initiated and completed by January 2025. The study uses anonymized intraoral photographs extracted from an institutional database; all patients provided written consent for the scientific use of their data, and all images were anonymized prior to analysis. Labeling, model training, and refinements continued until July/August 2025, while manuscript writing was performed in parallel with the development and testing of the Ultralytics YOLOv11 algorithm [14].

High-resolution intraoral photographs comprising five standard views (frontal occlusion; right and left buccal occlusion; maxillary and mandibular occlusal) were included, while syndromic conditions, cleft lip/palate, partial or complete edentulism, any active fixed orthodontic appliances or removable prostheses, and images with insufficient sharpness or illumination were excluded. File names were replaced with generic identifiers, and all EXIF metadata was removed; no facial images were used. Anonymized images were imported into a locally deployed Label Studio [15] instance for efficient manual annotation.

**Figure 1:**
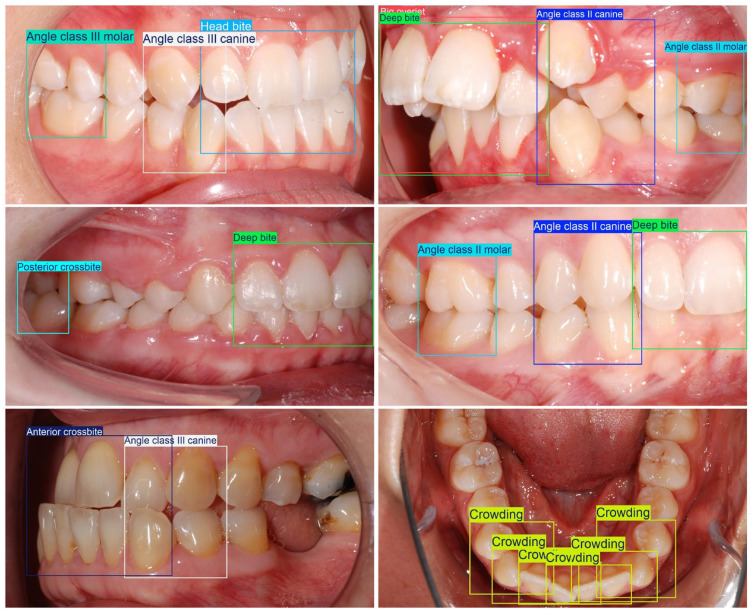
Labeling examples.

Seventeen malocclusion categories were prespecified a priori by an interdisciplinary team consisting of two orthodontists (clinical domain experts) and one computer-vision specialist before any dataset labeling was initiated. The selection and definition of these categories were based on the Index of Orthodontic Treatment Need (IOTN) [16]. In a joint consensus process, the team translated each clinical construct into operational, photograph-adapted bounding box rules that are clinically interpretable in 2D intraoral photographs (Table 1). In addition, all malocclusion classes were summarized in a separate overview table using partially simplified clinical descriptions intended to improve readability; note that these simplified descriptions may not fully match formal orthodontic terminology in every nuance (Table 2). A single orthodontically trained annotator (final-year postgraduate in orthodontics) subsequently labeled the complete dataset, strictly according to the written protocol (Figure 1).

Images with no target malocclusion were retained as negative-only images (background) and intentionally left without bounding boxes to support model calibration and reduce false positives. Ambiguous or borderline cases were not decided ad hoc; instead, they were escalated and adjudicated in consultation with the senior supervising orthodontist to ensure adherence to the prespecified definitions. Images without any of the predefined target malocclusions—including physiologic sagittal relations (Angle Class I) and physiologic overjet/overbite—were intentionally retained as negative-only background images and left without bounding boxes. This design provides the detector with explicit “no target finding” examples, supports probability calibration, and helps reduce false-positive detections. In total, 1220 background images were incorporated into the training process, comprising 189 frontal occlusion views, 358 left and right buccal occlusion views, and 673 maxillary and mandibular occlusal views. Excluding background images, there is an average of 2.94 bounding boxes per image.

In the approach, the dental malocclusion classes were treated as distinct objects within the intraoral photographs. For the detection of malocclusions, YOLOv11 [14,17] was used, which is a state-of-the-art real-time object detection model. YOLOv11 is an optimized single-stage object detector with maximized efficiency without compromising accuracy. Compared to earlier YOLO variants, it leverages an advanced CNN architecture with enhanced feature extraction layers (including more compute-efficient convolutional blocks and lightweight attention-based feature refinement) and an optimized detection head for precise bounding box regression, which motivated its selection for detecting visually subtle findings under variable intraoral image conditions. The YOLOv11 architecture consists of three main components: backbone, neck, and head. The backbone is composed of convolutional layers and acts as the basic feature extractor that transforms images into multi-scale feature maps. The neck component enhances and aggregates features across different scales. Finally, the head component predicts bounding boxes and class labels for localizing and classifying malocclusions. Key features of the architecture are efficient bottleneck blocks, a fast spatial pyramid pooling variant, and spatial attention mechanisms to capture rich feature representations. A detailed description of the key architectural components is given by Khanam and Hussain [17].

The object detection model was not trained from scratch, but instead, a pretrained network initialized on the COCO dataset was employed. The Ultralytics YOLO11 framework [14] provides various pretrained models of different scales, which differ in depth and width. To achieve a balanced trade-off between detection accuracy and computational efficiency, the medium-scale variant was selected. Furthermore, model robustness and generalization capabilities were enhanced by applying a sophisticated data augmentation strategy to the training data. The strategy included, for example, horizontal flipping, random erasure, mosaic augmentation, and color space augmentations, such as random adjustments of hue, saturation, and value, as well as geometric transformations like rotations, translations, scaling, and perspective distortions combined with automated augmentation policies using RandAugment [18] with its default settings, as this configuration was empirically optimized across diverse datasets.

Overall, the model was fine-tuned for a total number of 100 epochs using the AdamW optimizer of the YOLO11 framework [14] with an initial learning rate of 0.000526, a 3-epoch learning rate warm-up, a momentum of 0.9, and a batch size of 16. During preprocessing, the images are resized such that the longest side of the image is scaled to 960 pixels while maintaining the aspect ratio. This training process took approximately 3 h using an NVIDIA L40S GPU (NVIDIA Corporation, Santa Clara, CA, USA) on a GPU server with an AMD EPYC 9554P 64-Core CPU and 768 GB RAM.

## 3. Results

In the following data statistics, the evaluation metrics used and the evaluation results are presented. In addition to the overall and class-wise accuracies, the precision–recall curves as well as the view-wise patterns are analyzed.

### 3.1. Data

Due to an insufficient number of training and test samples, the malocclusion classes Transposition and Non-occlusion were removed from the dataset. The remaining dataset comprises 5854 intraoral photographs across five standard views (frontal occlusion; right/left buccal; maxillary and mandibular occlusal), yielding 13,628 annotated instances across 15 malocclusion classes. This dataset was divided into a stratified training and test set. While the training set contains 5364 images with 12,301 instances, the independent test set contains 490 images and 1327 instances/bounding boxes. Table 3 provides an overview of the corresponding class distributions.

### 3.2. Evaluation Metrics

To summarize the overall malocclusion detection accuracy of the YOLOv11 model, the mean average precision score at an intersection–over-union (IoU) threshold of 50% (mAP50) was used. This is a widely used metric to evaluate and compare object detection approaches. Per malocclusion class, average precision (AP) calculates the area under the precision–recall curve, where detections are considered as true positives if the predicted bounding box overlaps with the groundtruth bounding box by at least 50%. The mean of the class-wise AP values is the final mAP50 score. Furthermore, precision and recall values are provided. On the one hand, precision measures the proportion of correctly predicted bounding boxes among all predicted bounding boxes. It reflects the model’s ability to avoid false positives. On the other hand, recall quantifies the proportion of objects correctly detected by the model among all objects present in the dataset. It reflects the model’s ability to find all relevant instances. The presented precision and recall values are calculated at the threshold given by the maximum F1 score for each class, where the F1 score is the harmonic mean of precision and recall. The mean of the class-wise recall and precision values, respectively, are the macro recall (macro-R) and macro precision (macro-P) scores.

### 3.3. Overall Accuracy and Class-Wise Accuracy on the Test Set

Across the 15 evaluated malocclusion classes, the YOLOv11 detector achieved an overall accuracy of 87.8% mAP50. The macro precision and recall scores are 76.9% and 86.1%, respectively (see Table 4).

To enable a more nuanced analysis of the class-wise AP50 scores, the value range was subdivided into three accuracy levels/categories: high (≥90%), medium (80–90%), and low (<80%). While the majority of malocclusion classes (Deep bite, Diastema, Angle class II canine, Anterior open bite, Midline shift, Angle class II molar, Spacing, and Crowding) achieved high AP50 scores, Anterior crossbite, Angle class III molar, Head bite, and Posterior open bite fell into the medium category. Lower or moderate AP50 scores were obtained for Angle class III canine, Posterior crossbite, and Big overjet. Per-class precision/recall patterns match these bands (e.g., Deep bite and Diastema show high precision and recall; Posterior crossbite and Big overjet are comparatively lower) (see Table 4).

**Figure 2:**
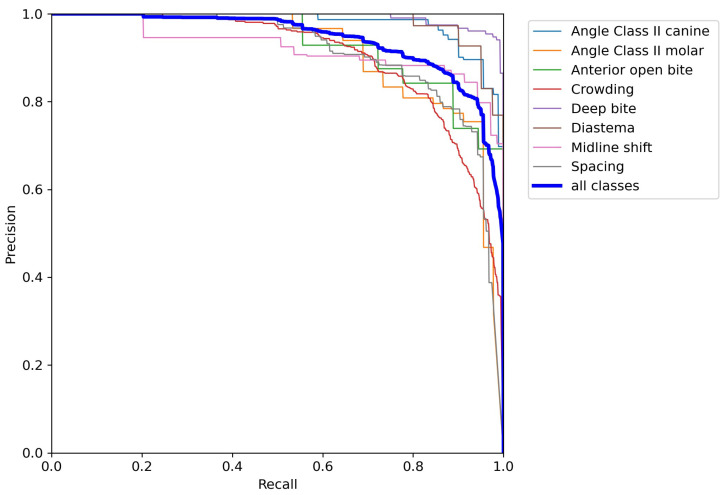
Precision–recall curves of the classes with high accuracy level.

**Figure 3:**
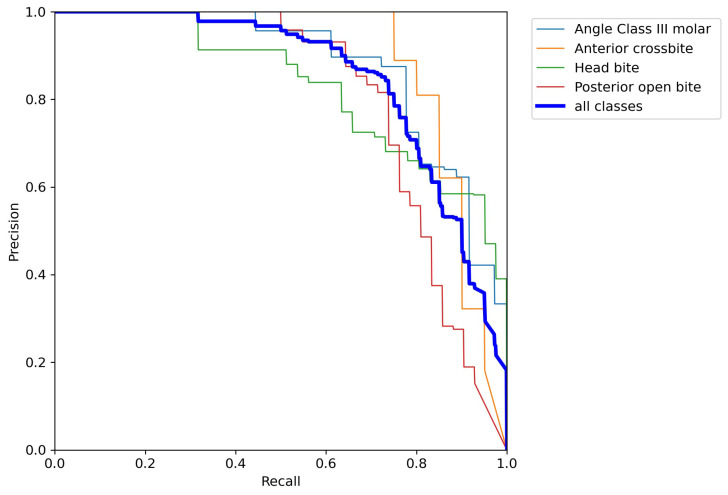
Precision–recall curves of the classes with medium accuracy level.

**Figure 4:**
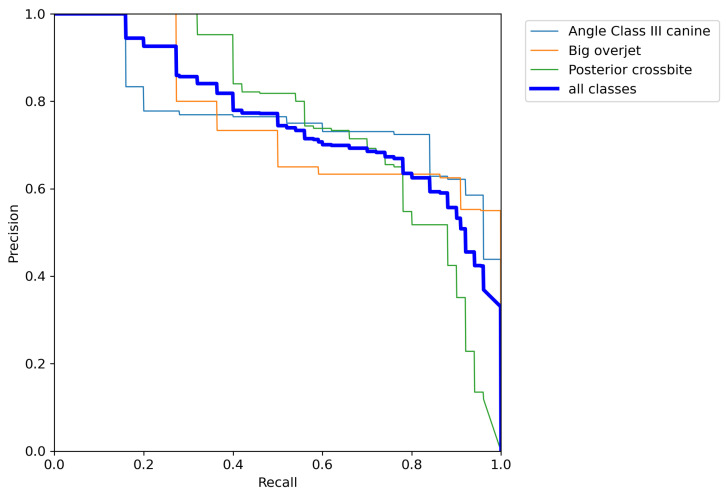
Precision–recall curves of the classes with low accuracy level.

The precision–recall curves in Figure 2, Figure 3 and Figure 4 are grouped according to the three accuracy levels. They illustrate the trade-off between the model’s ability to find all relevant malocclusions (recall) and its accuracy in predicting only correct examples (precision). Most of the precision–recall curves of the proposed model exhibit a high precision (above 90%) for recall values up to about 70%, indicating that the detector is highly reliable within this recall range. While Posterior crossbite, Big overjet, and Angle Class III canine exhibit earlier precision drop-off as recall increases, the curves for Deep bite, Diastema, and Angle Class II canine/molar remain near the top-right corner, showing strong performance over most thresholds.

Inference runtimes were measured on an NVIDIA L40S GPU (7.4 ms per image) and an AMD EPYC 9554P 64-Core CPU (226.1 ms per image), showing a high inference speed-up on GPU hardware.

### 3.4. View-Wise Patterns

Label distributions (Table 5) largely follow landmark visibility. Sagittal classes (angle classifications) are annotated almost exclusively on buccal views (>99% of labels), while midline shift is nearly confined to the frontal view (~99%). Arch-level traits (Crowding, Spacing) concentrate on occlusals (e.g., crowding 0/4/5628; spacing 0/7/1654 for frontal/buccal/occlusal). A small residual of Angle and Overjet labels on frontal images likely reflects slight camera yaw/tilt exposing canine–molar steps or incisal projection.

Performance by view (Table 6) mirrors this alignment. Buccal views yield high AP50 for Angle II canine/molar (97.5/91.1%) and for Anterior open bite (97.2%). Occlusals dominate arch-level traits (Spacing 91%; Crowding 90.1%). Frontal views best capture midline shift (91.9%), Deep bite (99.3%), and Diastema (97.8%). Two exceptions are instructive: (i) Big overjet is weak on frontal (49.7%) but improves on buccal (77.4%), suggesting reliance on sagittal profile cues; (ii) Posterior crossbite achieves higher AP50 on frontal (82%) than on buccal (72%) despite more buccal labels, which is consistent with transverse clues visible in frontal (e.g., unilateral buccal corridor narrowing, occlusal cant). Posterior open bite is modest overall and notably lower on frontal (69.8%) versus buccal (84.3%), indicating a dependence on posterior contact visualization.

These trends are reflected in precision–recall behavior and in the class-wise operating-point metrics. Anterior/frontal-occlusal traits showed consistently strong performance (e.g., Deep bite: precision/recall 95.2%/97.7%; Diastema: 88.4%/95%; Angle Class II canine: 89.2%/92%), whereas posterior/transverse classes were more error-prone (Posterior crossbite: 71%/66%; Posterior open bite: 69.4%/75.4%). Notably, several classes exhibited a predominantly false-positive tendency (lower precision), including Big overjet (62.2%), Head bite (63%), and Anterior open bite (66.6%; recall 100%), which is consistent with earlier precision drop-off as recall increases. In contrast, predominant false negatives (lower recall) were most pronounced for Posterior crossbite (recall 66%) and, to a lesser extent, Angle Class III molar (77.8%), supporting the notion that posterior/transverse assessment is more dependent on buccal/posterior visibility in 2D views. Overall mean precision/recall was 76.9%/86.1%.

## 4. Discussion

We evaluated a YOLOv11 one-stage detector for localizing and classifying 15 malocclusion categories across five routine intraoral views (frontal occlusion; right/left buccal; maxillary/mandibular occlusal). The model was consistently strong on visually salient anterior traits and more variable on posterior and sagittal categories. In clinical terms, midline deviations, diastemas, anterior open bite, and related spacing features were recognized reliably, whereas crossbites and Angle Class III patterns were detected less consistently. This pattern aligns with the visibility and consistency of routine photographs: anterior landmarks are usually well-captured in frontal and occlusal views, while posterior regions are prone to partial coverage, shadowing, and slight angulation differences that obscure cusp relationships [19,20,21].

Moreover, part of the imbalance could be view-conditioned: Table 5 shows several classes with non-trivial label counts in views where visual evidence is intrinsically weaker, which aligns with depressed per-view AP50 in Table 6. For example, Big overjet is poorly learned on frontal occlusion (AP50 = 49.7%) but improves on buccal (77.4%), and Posterior open bite shows a similar pattern (69.8% frontal vs. 84.3% buccal). By contrast, Posterior crossbite is slightly stronger on frontal than buccal (82% vs. 72%), suggesting frontal transverse cues can sometimes compensate, but likely with higher variance. Such label–view mismatches inject conditional label noise and exacerbate minority class effects, lowering overall accuracy and calibration. Mitigations include considering different views in the training and inference phase using, e.g., weighting schemes or ensemble strategies.

**Figure 5:**
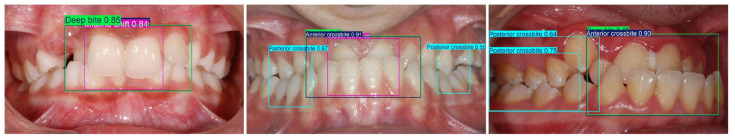
Object detection results for Anterior crossbite and Deep bite.

**Figure 6:**
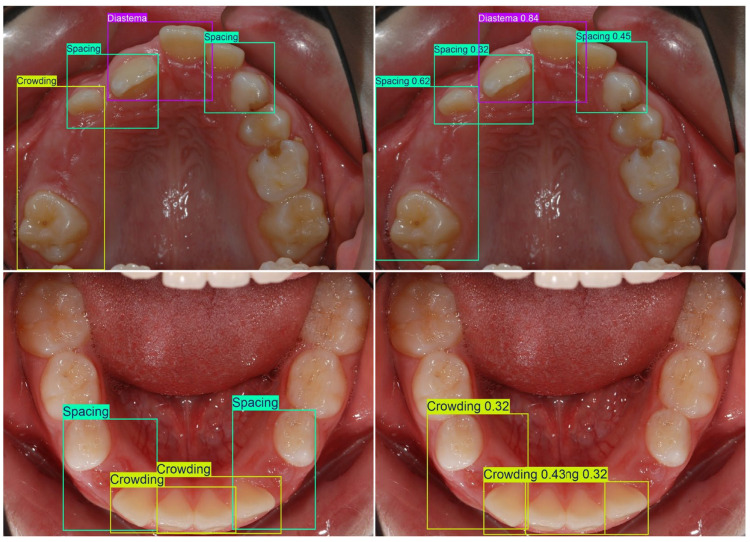
Spacing crowding confusion: (**left**) groundtruth and (**right**) malocclusion detection results.

Class imbalance likely amplified these effects because rarer traits provided fewer learning examples and ambiguous presentations, such as combined anterior crossbite with deep bite (Figure 5) or eruptive crowding that can mimic spacing (Figure 6), and added uncertainty. These phenomena are well-described in imbalanced medical imaging and noisy label settings, where minority classes and borderline phenotypes degrade classifier calibration and recall [22,23].

The absence of calibrated measurements (e.g., millimeters of overjet/overbite) further pushes borderline cases to rely on visual cues alone. Integrating quantitative modules is feasible: recent work using intraoral photographs estimated occlusal categories and continuous overjet/overbite values, showing that adding quantitative targets can strengthen decision rules for borderline cases [24].

These characteristics are relevant for several clinical use cases. High accuracy on prominent anterior traits suggests that a photograph-based detector could assist with pre-screening referrals from general and pediatric dentists, flagging obvious malocclusions for specialist review; related remote triage workflows using parent- or patient-captured smartphone images have shown acceptable feasibility and diagnostic signals in controlled settings [25].

Automated overlays could also enhance documentation by marking regions of interest during appointments to support consistent records and longitudinal monitoring aligned with emerging occlusion classification systems trained directly on intraoral photos [26,27].

Such tools should remain adjuncts, not replacements, for comprehensive diagnosis: 2D photographs capture primarily dento-alveolar relationships and can miss functional, skeletal, or transverse discrepancies that still require clinical examination, study models, and (when justified) radiographs [28].

Data quality and representation remain central. Our single-center dataset and persistent class imbalance for posterior anomalies limit generalizability. Robust performance will require larger, better-balanced, multi-center datasets spanning diverse operators and imaging conditions, potentially via decentralized learning to enable multi-site collaboration without sharing raw data [29]. Because malocclusions are inherently three-dimensional and view-dependent, combining information across views is a logical next step: traits such as diastema or midline shift are best seen frontally or on occlusal images, while sagittal relationships depend on buccal views. Cross-view and multimodal feature fusion (e.g., learning across multiple 2D views or combining photos with 3D scans/CBCT/cephalometrics) can improve recall and reduce misclassifications [30,31].

Annotation practice is equally pivotal. Explicit, class-specific bounding box guidelines were used, yet real-world cases blur category boundaries. To reduce label noise, future datasets should consider multi-reader consensus and adjudication workflows, or algorithms that learn from multiple annotators and model annotator variance approaches shown to improve robustness on ambiguous medical images [32,33].

Photographic factors also matter. Underperformance on posterior crossbite reflects the influence of camera angle, exposure, and field of view. Practical mitigations include aligning the occlusal plane horizontally, ensuring adequate lip retraction, careful cropping, and manual focusing; ring flashes or diffusers can stabilize contrast; smartphone holders/guides help align to the occlusal plane; and automated quality-control filters can flag inadequate images (e.g., blurred or poorly centered views) before model inference [34].

Rare malocclusions remain a challenge. Addressing underrepresented classes will likely require collaborative data sharing, targeted case collection, and carefully validated synthetic augmentation. Dental imaging studies have shown that GAN/diffusion models can generate realistic intraoral or panoramic images and boost performance on scarce classes, provided transparency about dataset composition and failure analyses [35,36].

### Limitations of the Study

**Figure 7:**
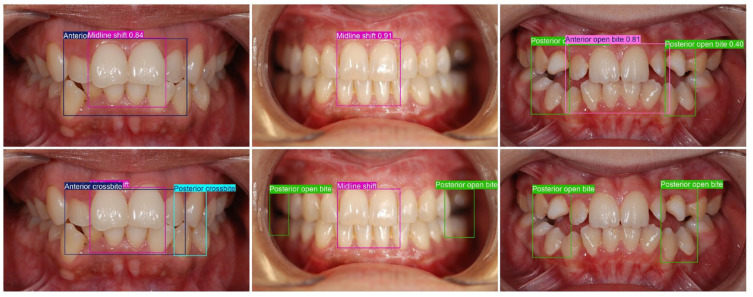
Malocclusion detection results with false positives/negatives: (**top**) predictions and (**bottom**) groundtruth.

Finally, several limitations frame future work. The dataset was derived from a single center. All annotations were produced by a single rater, so inter-rater variability was not assessed, and labeling bias cannot be excluded. Beyond mAP50, a macro precision of 76.9% and a macro recall of 86.1% were reported (Table 4). This indicates that (averaged across classes) most annotated findings were retrieved, while roughly one-quarter of predicted detections represented false positives. The observed class-specific precision–recall patterns likely reflect inherent constraints of 2D intraoral photography and operational labeling, particularly in borderline cases. Posterior/transverse findings depend strongly on buccal/posterior visibility and consistent intercuspation, and limited posterior exposure in single views can therefore increase missed detections (e.g., Posterior crossbite, recall 66%). In contrast, continuous anterior traits are especially sensitive to perspective, camera angle, and mild presentations, which can promote overcalling and thus reduce precision (e.g., Big overjet 62.2%; Head bite 63%; Anterior open bite 66.6% with recall 100%). These view- and definition-dependent failure modes are also exemplified by ambiguous cases in Figure 7 (left to right): (i) a posterior crossbite evident across views was not detected on the frontal image, possibly because the transverse relationship is difficult to judge from that angle, even for trained specialists; (ii) a posterior open bite was missed on a frontal image, which is consistent with limited visibility and with Table 6 showing lower performance for frontal versus lateral views (AP50 69.8% vs. 84.3%); and (iii) the model correctly detected a posterior open bite on the frontal view but additionally predicted an anterior open bite, an arguably “pattern-correct” output that conflicts with our operational labeling rule (only teeth 12 and 22 lacked vertical overlap), suggesting that separate labeling rules (or an additional category) for an open bite/vertical tendency may be needed. Importantly, resolving such borderline vertical diagnoses may require complementary records (e.g., lateral cephalometry), highlighting a bottleneck of photo-only assessment and reinforcing the rationale for cross-view and potentially multimodal data fusion to approach higher clinical accuracy. Overall, variability in acquisition conditions (retraction, lighting, and anatomy) likely contributes further to class-dependent false positives/false negatives. In addition, we have not yet conducted external or prospective validation, nor evaluated end-to-end workflow integration in routine practice. Advancing beyond this study will require multi-center, multi-reader datasets; strategies to address class imbalance; cross-view and potentially multimodal fusion to improve performance in visually constrained regions; quantitative measurement modules to move beyond categorical outputs where appropriate; and pragmatic image-quality controls and standardization to improve robustness in real-world acquisition conditions.

## 5. Conclusions

A YOLOv11-based model trained on standardized intraoral photographs can accurately detect several malocclusion categories and may serve as a scalable decision-support tool in orthodontic workflows. This study presents one of the first multi-class, multi-view object detectors for orthodontic photography trained with explicit bounding box definitions, achieving a high mean AP with particularly strong performance on anterior traits; conversely, lower performance on posterior and sagittal anomalies highlights priorities for subsequent phases addressing class imbalance, improving posterior visualization, and enabling multi-center validation. Placing these findings within the broader dental-AI literature underscores the importance of standardized imaging protocols, balanced and diverse datasets, clear, reproducible annotation guidelines, and multimodal integration. Future work should expand datasets across institutions, incorporate quantitative measures (e.g., overjet/overbite) and additional modalities, and rigorously test detectors prospectively in clinical workflows to move towards reliable and generalizable AI-assisted orthodontic screening.

## Figures and Tables

**Table 1:** Labeling guidelines.

Malocclusion (Classification)	Labeling Guidelines/Bounding Box (BB) Instructions
Angle Class II molar, Angle Class III molar	BB over the first maxillary and mandibular molar
Angle Class II canine, Angle Class III canine	BB over the affected upper and lower canines, as well as the first lower premolar
Crowding	BB over the affected tooth as well as the two adjacent teeth
Spacing	BB over the existing gap as well as the two adjacent teeth
Transposition	BB over the affected teeth
Big overjet, Anterior crossbite, Deep bite, Head bite, Anterior open bite	BB over the four upper incisors as well as the overlapping lower canines and incisors
Posterior open bite, Posterior crossbite, Non-occlusion	BB over the affected posterior teeth
Midline shift	BB over the upper central incisors as well as the overlapping lower incisors and canines
Diastema	BB over the upper central incisors

**Table 2:** Explanation of the malocclusions (classifications).

Malocclusion (Classification)	Definition of the Malocclusion
Angle Class II canine	The maxillary canine cusp tip is positioned mesial/anterior to the ideal embrasure between the mandibular canine and 1st premolar
Angle Class II molar	The mesiobuccal cusp of the maxillary 1st molar is anterior/mesial to the buccal groove of the mandibular 1st molar
Angle Class III canine	The maxillary canine cusp tip is positioned distal to the ideal embrasure between the mandibular canine and 1st premolar
Angle Class III molar	The mesiobuccal cusp of the maxillary 1st molar is posterior/distal to the buccal groove of the mandibular 1st molar
Anterior crossbite	One or more maxillary incisors occlude lingual/palatal to the mandibular incisors
Anterior open bite	No vertical overlap of anterior teeth in occlusion; a visible gap remains between upper and lower incisors when posterior teeth are in contact
Big overjet	Increased horizontal overlap: maxillary incisors are clearly far ahead of mandibular incisors (photo-based; in our case, that would correspond to more than 5 mm clinically)
Crowding	Insufficient space in the arch, causing overlapping, rotations, or displacement of teeth
Deep bite	Mandibular incisors are covered too much by maxillary incisors (>50% coverage)
Diastema	A distinct midline gap between the maxillary central incisors, beyond normal contact separation
Head bite	Incisal edge-to-edge relationship: upper and lower incisors meet edge-to-edge with ~0 overjet
Midline shift	The upper and lower dental midlines do not coincide (visible mismatch between the contact points of the central incisors)
Posterior crossbite	One or more posterior teeth occlude in reverse transverse relationship: maxillary posterior teeth are positioned lingual/palatal to mandibular posterior teeth
Posterior open bite	No occlusal contact in the posterior segment despite attempted occlusion; a visible vertical gap between posterior antagonists
Spacing	When no contact point was present and gingiva was visible between the teeth; midline diastema was excluded
Transposition	Two teeth exchange positions in the arch
Non-occlusion	Non-intercuspation due to transverse “pass-by”: posterior teeth do not occlude because the maxillary segment passes buccally or lingually to the mandibular antagonists (e.g., scissor/Brodie-type non-occlusion)

**Table 3:** Class-wise statistics of images and instances in the training and test datasets for the 15 malocclusion classes.

	Training Set		Test Set	
Category	Images	Instances	Images	Instances
All	5364	12,301	490	1327
Angle Class II canine	828	828	90	90
Angle Class II molar	372	372	45	45
Angle Class III canine	225	225	25	25
Angle Class III molar	327	327	36	36
Anterior crossbite	177	177	20	20
Anterior open bite	210	210	18	18
Big overjet	181	181	22	22
Crowding	1235	5087	133	545
Deep bite	1173	1174	128	128
Diastema	334	340	38	40
Head bite	352	353	41	41
Midline shift	684	684	69	69
Posterior crossbite	400	459	44	50
Posterior open bite	331	379	37	42
Spacing	588	1505	66	156

**Table 4:** Class-wise malocclusion detection results in terms of recall, precision, and AP50 on the test set.

Class	Precision	Recall	AP
Angle Class II canine	0.892	0.920	0.975
Angle Class II molar	0.781	0.870	0.911
Angle Class III canine	0.679	0.840	0.760
Angle Class III molar	0.837	0.778	0.874
Anterior crossbite	0.800	0.850	0.883
Anterior open bite	0.666	1.000	0.928
Big overjet	0.622	0.824	0.753
Crowding	0.779	0.848	0.901
Deep bite	0.952	0.977	0.988
Diastema	0.884	0.950	0.979
Head bite	0.630	0.829	0.827
Midline shift	0.797	0.969	0.918
Posterior crossbite	0.710	0.660	0.756
Posterior open bite	0.694	0.754	0.802
Spacing	0.815	0.853	0.910
Mean	0.769	0.861	0.878

**Table 5:** View-wise groundtruth label distribution in the overall dataset regarding the following three view categories: frontal occlusion, left and right buccal occlusion, and maxillary and mandibular occlusal.

Category	Frontal Occlusion	Left and Right Buccal Occlusion	Maxillary and Mandibular Occlusal
Angle Class II canine	2	916	0
Angle Class II molar	2	415	0
Angle Class III canine	0	250	0
Angle Class III molar	0	363	0
Anterior crossbite	71	126	0
Anterior open bite	88	140	0
Big overjet	9	194	0
Crowding	0	4	5628
Deep bite	411	891	0
Diastema	211	4	165
Head bite	146	247	0
Midline shift	748	5	0
Posterior crossbite	222	287	0
Posterior open bite	110	311	0
Spacing	0	7	1654

**Table 6:** Class-wise malocclusion detection results in terms of AP50 on different subsets of the test set. The subsets are the three view categories: frontal occlusion, left and right buccal occlusion, and maxillary and mandibular occlusal.

Category	Frontal Occlusion	Left and Right Buccal Occlusion	Maxillary and Mandibular Occlusal
Angle Class II canine	-	0.975	-
Angle Class II molar	-	0.911	-
Angle Class III canine	-	0.760	-
Angle Class III molar	-	0.876	-
Anterior crossbite	0.848	0.886	-
Anterior open bite	0.897	0.972	-
Big overjet	0.497	0.774	-
Crowding	-	-	0.901
Deep bite	0.993	0.986	-
Diastema	0.978	-	0.988
Head bite	0.823	0.842	-
Midline shift	0.919	-	-
Posterior crossbite	0.820	0.720	-
Posterior open bite	0.698	0.843	-
Spacing	-	-	0.910

## Data Availability

The data presented in this study are not publicly available due to institutional regulations, but it may be made available from the corresponding author upon reasonable request.

## Abbreviations

The following abbreviations are used in this manuscript:

2D	Two-dimensional
3D	Three-dimensional
AI	Artificial intelligence
AP	Average precision
AP@0.5	Average precision at IoU 0.5
AdamW	Adam optimizer with decoupled weight decay
CBCT	Cone-beam computed tomography
CNN	Convolutional neuronal network
COCO	Common objects in context (dataset)
EXIF	Exchangeable image file format
F1	Harmonic mean of precision and recall
FN	False negative
FP	False positive
GAN	Generative adversarial network
IoU	Intersection over union
IOTN	Index of Orthodontic Treatment Need
mAP	Mean average precision
mAP50	Mean average precision at IoU 0.5
ML	Machine learning
P	Precision
PR	Precision–recall
R	Recall
TP	True positive
YOLO	You Only Look Once
YOLOv11	You Only Look Once, version 11
